# Polyacrylamide Ferrogels with Magnetite or Strontium Hexaferrite: Next Step in the Development of Soft Biomimetic Matter for Biosensor Applications

**DOI:** 10.3390/s18010257

**Published:** 2018-01-16

**Authors:** Alexander P. Safronov, Ekaterina A. Mikhnevich, Zahra Lotfollahi, Felix A. Blyakhman, Tatyana F. Sklyar, Aitor Larrañaga Varga, Anatoly I. Medvedev, Sergio Fernández Armas, Galina V. Kurlyandskaya

**Affiliations:** 1 Institute of Natural Sciences and Mathematics, Ural Federal University, Ekaterinburg 620002, Russia; safronov@iep.uran.ru (A.P.S.); emikhnevich93@gmail.com (E.A.M.); Feliks.Blyakhman@urfu.ru (F.A.B.); t.f.shkliar@urfu.ru (T.F.S.); medtom@iep.uran.ru (A.I.M.); 2 Institute of Electrophysics, Ural Division RAS, Ekaterinburg 620016, Russia; 3 Departamento de Electricidad y ElectrónicaUniversidad del País Vasco UPV/EHU, 48080 Bilbao, Spain; lotfollahi@gmail.com; 4 Deapartment of Physics, University of Birjand, Birjand 97175-615, Iran; 5 Biomedical Physics and Engineering Department, Ural State Medical University, Ekaterinburg 620028, Russia; 6 Advanced Research Facilities (SGIKER), Universidad del País Vasco UPV-EHU, 48080 Bilbao, Spain; aitor.larranaga@ehu.eus (A.L.V.); sergio.fernandez@ehu.eus (S.F.A.)

**Keywords:** magnetic nanoparticles, strontium hexaferrite, magnetite, ferrofluids, polyacrylamide gel, ferrogel, tissue engineering, magnetic biosensors, giant magnetoimpedance

## Abstract

Magnetic biosensors are an important part of biomedical applications of magnetic materials. As the living tissue is basically a “soft matter.” this study addresses the development of ferrogels (FG) with micron sized magnetic particles of magnetite and strontium hexaferrite mimicking the living tissue. The basic composition of the FG comprised the polymeric network of polyacrylamide, synthesized by free radical polymerization of monomeric acrylamide (AAm) in water solution at three levels of concentration (1.1 M, 0.85 M and 0.58 M) to provide the FG with varying elasticity. To improve FG biocompatibility and to prevent the precipitation of the particles, polysaccharide thickeners—guar gum or xanthan gum were used. The content of magnetic particles in FG varied up to 5.2 wt % depending on the FG composition. The mechanical properties of FG and their deformation in a uniform magnetic field were comparatively analyzed. FG filled with strontium hexaferrite particles have larger Young’s modulus value than FG filled with magnetite particles, most likely due to the specific features of the adhesion of the network’s polymeric subchains on the surface of the particles. FG networks with xanthan are stronger and have higher modulus than the FG with guar. FG based on magnetite, contract in a magnetic field 0.42 T, whereas some FG based on strontium hexaferrite swell. Weak FG with the lowest concentration of AAm shows a much stronger response to a field, as the concentration of AAm governs the Young’s modulus of ferrogel. A small magnetic field magnetoimpedance sensor prototype with Co_68.6_Fe_3.9_Mo_3.0_Si_12.0_B_12.5_ rapidly quenched amorphous ribbon based element was designed aiming to develop a sensor working with a disposable stripe sensitive element. The proposed protocol allowed measurements of the concentration dependence of magnetic particles in gels using magnetoimpedance responses in the presence of magnetite and strontium hexaferrite ferrogels with xanthan. We have discussed the importance of magnetic history for the detection process and demonstrated the importance of remnant magnetization in the case of the gels with large magnetic particles.

## 1. Introduction

Biomedical application of magnetic materials is a rapidly extending area, which requires additional research [1,2,3]. Over the past decade, many efforts have been made for the development of a generation of magnetic biosensors, i.e., compact analytical devices incorporating a biological sensitive element, integrated in a physicochemical transducer employing a magnetic field [4,5]. These devices are becoming common tools used for a variety of biomedical applications [6,7,8]. In many cases, magnetic nanoparticles (MNPs) work as biomolecular labels [4,7] which must be provided in the form of water-based ferrofluids [2,3,9]. Apart from evaluating the concentration of the magnetic labels in a test solution, the detection of superparamagnetic nanoparticles (MNPs) after intracellular uptake was also tested [6,10]. However, one of the greatly requested applications for different cancer therapies (the detection of the MNPs incorporated into living tissues) or regenerative medicine has not yet been properly addressed. One of the fundamental reasons for this delay is the sensitivity of the existing sensing devices. Giant magnetoimpedance (MI) had attracted special attention as the phenomenon providing the basis for sensors capable of detecting picotesla magnetic fields [11,12]. The great advantage of the present day available MI-sensitive elements is their easily approached enhanced sensitivity of the order of 100%/Oe for MI ratio variation [13].

The idea of using a magnetic field sensor in combination with magnetic particles/nanoparticles working as magnetic markers for the detection of molecular recognition events was first reported in 1998 by Baselt et al. [4]. Such a device was based on giant magnetoresistance (GMR) technology and employed magnetic composite microbeads for simultaneous characterization of many biomolecular interaction events. Different geometry was proposed for a magnetoresistive biosensor prototype designed for the detection of a single micro magnetic sphere by a ring-shaped element working on anisotropic magnetoresistance effect [14]. The important disadvantage of exchange-coupled GMR sensors is the high field required for a reasonable resistance change. Microsized spin valve sensors with lower operation fields were also developed for detection of biomolecules with magnetic markers [15]. Another approach employed the Hall effect for a sensor based on standard metal-oxide-semiconductor technology for selective detection of magnetic markers [16] (Besse et al. 2002). With respect to the magnetic field sensitivity, the MI effect is the best option for the creation of magnetic biosensors: it can be mentioned that the maximum sensitivity achieved at present is ~2%/Oe for GMR materials [17].

There were attempts to develop MI-biosensors based on sensitive elements of different types: rapidly quenched wires, glass-coated microwires, amorphous ribbons and thin films [5,6,18,19,20]. Different MI-materials have different advantages and disadvantages, summarized in various topical reviews [12,20,21]. Although, thin films were under special focus recently due to their excellent compatibility with semiconductor electronics [5,22,23,24], cheap MI-biosensors with a disposable sensitive element in the form of a stripe are being developed. These disposable sensors can be used by non-skilled personnel in non-sterile environments. Amorphous Co-based rapidly quenched ribbons are excellent candidates in this case [10,13,25,26].

The development of MI biosensor to evaluate the properties of biological tissues is strongly conditioned by the availability of reliable samples. Biological materials present a wide variety of morphologies especially in the case of cancer affected tissues, characterized by the accelerated growth of irregular blood vessels [27]. In our previous works related to MI biosensors with thin film a sensitive element [5,28], we proposed to substitute biological samples at the first stage of the development of the MI biosensor prototype by the adequate model materials—synthetic ferrogels mimicking the main properties of the living tissues [29,30,31]. Those ferrogels were based on MNPs obtained by the electrophysical technique of laser target evaporation [32,33]. In addition, it is necessary to point out that a wide variety of morphologies of the cancer affected tissues become inevitably reflected in the corresponding wide varieties of their mechanical and magneto-electrical properties.

The choice of the MNPs was defined by an important condition for the majority of magnetic biosensing cases adapted to the magnetic label detection principle: the stray fields induced by the magnetic markers are employed as biomolecular labels providing a means for the transfer of information about the concentration of magnetic labels and therefore the biocomponent of interest [4,18]. The sensitivity limit is related to the type of MNPs—the magnetic moment of an individual particle in the external magnetic field governs the stray fields and the biodetection limit. The temptation to increase the magnetic moment of the individual magnetic label is strictly limited by the condition of superparamagnetic state [34,35] in order to avoid MNPs’ agglomeration in zero field. When the magnetic particles or MNPs become incorporated into a tissue and spatially localized, their size might be much larger as a result of non-proper functioning of the living system.

We therefore propose to study various kinds of ferrogels with micron sized commercially available magnetic particles (MPs) in order to create reliable samples mimicking natural tissue and evaluate the possibility of their detection by an amorphous Co-based ribbon sensitive element. Detection of the stray fields of magnetic particles incorporated into a living system have a number of additional requests. As the living tissue is basically a “soft matter.” the mechanical properties are important as well as possible deformations caused by the application of the external magnetic field. We describe our experience of the synthesis and characterization of magnetite Fe_3_O_4_ and strontium hexaferrite SrFe_12_O_19_ powder based ferrogels including the measurements of the change of MI of the Co-based ribbon sensitive element in the presence of ferrogels with different concentration of iron oxide magnetic particles using a specially designed MI sensor prototype as a model for biosensors.

## 2. Experimental

### 2.1. Materials 

For the preparation of ferrogels we used commercial magnetic powders of oxides: magnetite Fe_3_O_4_ (Alfa Aesar, Ward Hill, MA, USA) and strontium hexaferrite SrFe_12_O_19_ powder mark 28PFS250 (Olkon, Kineshma, Russian Federation).

**Figure 1:**
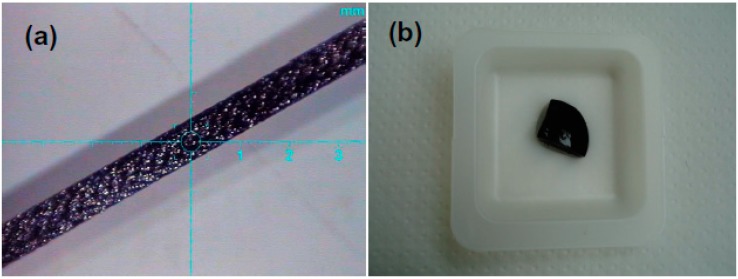
General view in optical microscope of the free surface of Co_68.6_Fe_3.9_Mo_3.0_Si_12.0_B_12.5_ rapidly quenched amorphous ribbon based MI sensitive element (**a**); general macroscopic view of xanthan-based ferrogel FG-2a with magnetite magnetic particles in 3.19 weight % concentration (**b**).

Amorphous ribbons with Co_68.6_Fe_3.9_Mo_3.0_Si_12.0_B_12.5_ nominal composition (Figure 1a) were prepared by rapid quenching technique [36] and all measurements were made in their as-quenched state, i.e., without additional heat treatments or surface modifications. The saturation of magnetostriction coefficient (λs), was estimated by the inductive technique measurements of the hysteresis loops of the ribbon at 1.7 Hz under different mechanical stresses. The magnetostriction coefficient was calculated from the slope of anisotropy field vs. mechanical stress curve [37]. The requirements for the magnetoimpedance tests in magnetic biosensing conditions are: reasonably high MI effect sensitivity with respect to applied magnetic field and good corrosion stability. The composition with a molybdenum addition was selected on the basis of our previous evaluation of the MI effect and corrosion stability. We previously evaluated the resistance of the amorphous ribbons of different compositions by the weight loss in solutions of 3.0 M of orthophosphorous acid (H_3_PO_3_) in an ultrasonic bath [38]. Figure 1a shows a general view of the surface of Co_68.6_Fe_3.9_Mo_3.0_Si_12.0_B_12.5_ amorphous ribbon. The geometry of the ribbon for magnetic and magnetoimpedance measurements was 18 × 0.78 × 0.026 (mm).

### 2.2. Synthesis of Ferrogels

The basic composition of ferrogels comprised the polymeric network of polyacrylamide (PAAm), which was synthesized by free radical polymerization of monomeric acrylamide (AAm) (AppliChem, Darmstadt, Germany) in water solution at three levels of concentration (1.1 M, 0.85 M and 0.58 M) to provide gels with varying elasticity. N,N’ methylenebisacrylamide (Merck, Schtuchardt, Germany) was used as a cross-linker. Its molar concentration to monomer was 1:100. Ammonium persulfate was used as an initiator in 5 mM concentration. Powdered magnetite and strontium hexaferrite were dispersed in the reaction mixture before the synthesis. To prevent the precipitation of the particles polysaccharide thickeners were added to the reaction mixture—guar gum and xanthan gum (Sigma-Aldrich, St. Louis, MO, USA). Average molecular weight of polysaccharides was determined by viscometry using Mark-Houwink constants K = 1.7 × 10^−6^, a = 1.14 for xanthan [39] and K = 3.7 × 10^−4^, a = 0.74 for guar [40]. The molecular weight was found 2.2 × 10^6^ (xanthan gum) and 1.6 × 10^6^ (guar gum).

Thus, the synthesis of ferrogels included the following steps. First, 1% water solutions of guar or xanthan by weight were prepared by vigorous stirring, followed by equilibration for 24 h at room temperature and filtered to remove macroscopic gel fraction. Guar and xanthan gum solutions are viscoelastic at room temperature and elevated temperatures due to the formation of the physical network of polysaccharide chains. Then the weighted amount of magnetic filler was dispersed under vigorous stirring in the solution of a thickener. Due to the physical network of a polysaccharide the particles did not precipitate. Then the monomer, the cross-linker and the initiator were added to the dispersion. The reaction mixture was stirred, poured into a cylindrical polyethylene mold and placed under argon (to prevent inhibition of the reaction by oxygen) into an oven at 90 °C for 1 h until the polymerization of PAAm was completed. The total volume of the reaction mixture in each synthesis was 4.5 mL.

The amount of magnetic material was 0.5 g. The series of ferrogels filled with magnetite were denoted FG-1 if the thickener was guar gum and FG-2 if the thickener was xanthan gum. The series of ferrogels filled with strontium hexaferrite will be denoted FG-3 (thickener–guar gum) and FG-4 (thickener–xanthan gum). In each series, ferrogels varied in the concentration of the monomer–acrylamide taken in synthesis. Its concentration is placed after a mark of the series, e.g., FG-1-0.85 stands for the ferrogel based on magnetite dispersed with guar gum thickener and AAm concentration in the reaction mixture was 0.85 M. After the synthesis, ferrogels were taken out of the molds and kept in the excess of distilled water for two weeks with daily water renewal to wash out the residual monomer, salts and linear PAAm olygomers. The equilibrium swelling ratio of the gels after their stabilization was determined by gravimetry. The weight m_0_ of a swollen piece of gel of ca 0.5 g was measured using an analytical balance (Mettler-Toledo MS104S, Columbus, OH, USA). Then it was dried in an oven at 90 °C down to the constant weight of the dry residue m_1_. The apparent swelling ratio (α) was calculated according to the equation:

(1) $$\alpha =\frac{m_{0}-m_{1}}{m_{1}}$$

The value of the swelling ratio was used for the calculation of the particle content in the swollen gel applying the equation:

(2) $$\vartheta =\frac{\gamma }{1+\alpha }$$

where *γ* stands for the weight fraction of particles in the dry residue within the total comprised particles and cross-linked dry PAAm. The value of *γ* was calculated based on the proportions between the particles, the monomer, the cross-linker and the thickener taken in the synthesis. The values of the swelling ratio of ferrogels and the weight fraction of magnetic particles in the swollen gel are given in Table 1.

Table 1 also gives a description of xanthan-based gels (without magnetic particles, with different amount of thickener) used for MI measurements. They are very important for subtraction of the corresponding signal from the ferrogel samples in the course of the evaluation of the average contribution of the stray fields of magnetic particles. Figure 1b shows general view of FG-2a ferrogel as an example. Synthesized gel samples keep their shapes constant for a very short time period. The changes of the shape for longer time periods are related to water loss and generally they can be described as shrinking the sample as a whole.

### 2.3. Methods

The specific surface area (S_sp_) of powders was measured by the low-temperature sorption of nitrogen (Brunauer-Emmett-Teller physical adsorption (BET)) using Micromeritics TriStar3000 analyzer.

The X-ray diffraction (XRD) studies were performed by operating at 40 kV and 40 mA the DISCOVER D8 (Bruker, Leiderdorp, The Netherlands) diffractometer using Cu-Kα radiation (λ = 1.5418 Å), a graphite monochromator and a scintillation detector. The magnetic particles or amorphous ribbon cut in various pieces were mounted onto a zero-background silicon wafer placed in a sample holder. A fixed divergence and anti-scattering slit were used. Bruker software TOPAS-3 with Rietveld full-profile refinement was employed for the quantitative analysis of all the diffractograms.

Additionally, the average size of coherent diffraction domains (D_XRD_) was estimated using the Scherrer approach in the case of magnetic particles [41]. The coefficient k in the Scherrer equation was taken as k = 0.9 and instrumental broadening of the peaks FWHM_instr_ = 0.1.

The shape and size of the magnetic particles were studied by field-emission scanning electron microscopy (SEM) in a JEOL JSM-7000 F, equipped with a Schottky type field-emission gun. Energy-dispersive X-ray spectroscopy studies of dry ferrogels were done by ETM3000 SEM of HITACHI (Tokyo, Japan).

Magnetic measurements of the hysteresis loops (M(H)) were carried out by a vibrating sample magnetometer (VSM, Lake Shore 7404, Westerville, OH, USA) in the ±1.8 kOe field range. Although, in some cases magnetic powders were not completely saturated, for the sake of simplicity we decided to assign the saturation magnetization value to the magnetization in the field of 18 kOe. Ferrogels were measured in a polycarbonate capsule following a specially developed protocol. A vibrating sample magnetometer and a conventional inductive technique were used to study the magnetic properties of the ribbons at room temperature.

The measurements of the Young modulus of ferrogels were performed using a specially designed laboratory setup. Cylindrical gel samples ~10 mm in length and ~10 mm in diameter were used for mechanical testing. The equipment of laboratory design was built around an optical system based on a digital camera and contained the following: a bath for the gel sample, a semiconductor force transducer, an electromagnetic linear motor for applying mechanical deformations, a semiconductor optical transducer for the gel sample length measurement in dynamics. The gel sample was clamped vertically between the livers of force transducer and linear motor. To produce the “compression-decompression” cycle of gel samples, the triangular axial deformations (*ε*) with a constant rate 0.5 mm/s and an amplitude of up to 10% of the initial length of samples were applied. Gel tension (*σ*) was calculated as the recorded force normalized by the cross-sectional area of the gel sample. Gel tension (*σ*) was calculated as the recorded force normalized by the cross-sectional area of the gel sample. In the course of deformation, the cross-section of samples was corrected based on the assumption of a gel’s constant volume under deformation (Poisson ratio is close to 0.5) [42]. The Young modulus (*E*) was determined as the slope of the *σ*(*ε*) dependence:

(3) tgβ=σ/ε=E

The deformation of ferrogels in a uniform magnetic field was studied using the magnetic system designed and constructed at JSC “URALREDMET” (Verkhnyaya Pyshma, Russian Federation). The design of a magnetic array used for the magneto deformation studies was described early [43]. The system comprised permanent NdFeB magnets assembled with magnetic conductors and provided a vertical uniform magnetic field of 0.420 ± 0.5% T in the central zone of 1 cm^3^ in volume. The magnitude of the magnetic field inside the array was determined with a Gaussmeter TKH_4 instrument (Maurer Magnetic AG Grüningen/Switzerland).

The optical cuvette with ferrogel sample was placed in the central zone of the magnetic system. The cylindrical sample of ca 5 mm both in height and in diameter was pinned onto a needle holder affixed to the bottom of the cuvette. The cuvette was filled with water and sealed with Parafilm to prevent the deswelling of the ferrogel due to evaporation. The dimension changes of the ferrogel along the field lines and across the field lines were monitored by an EVS color VEC_545_USB optical system equipped with a condensing lens.

### 2.4. Magnetoimpedance Measurements

**Figure 2:**
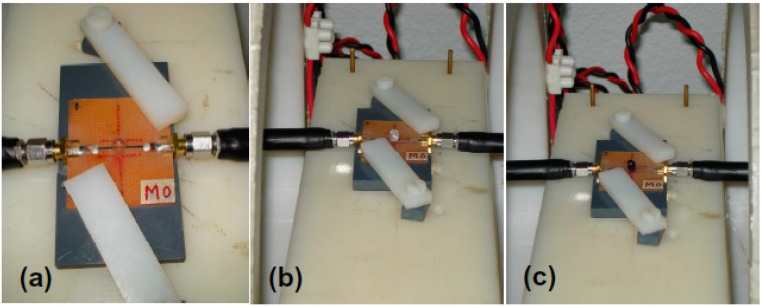
General view of the ribbon based MI sensitive element installed into “microstripe” line; external magnetic field is applied parallel to the long axis of the ribbon: (**a**) polymer capsule is empty (calibration measurement); (**b**) polymer capsule contains xanthan-based G-a gel without MPs; (**c**) polymer capsule contains xanthan-based FG-2a ferrogel with magnetite magnetic particles in 3.19 weight % concentration.

The length of the ribbon sensitive element for MI measurements was 18 mm (Figure 2). The ribbon was incorporated into a “microstripe” line (Figure 2a) and a uniform external magnetic field of up to 150 Oe was created by a pair of Helmholtz coils. The MI changes were calculated from the reflection S_11_ coefficient measured by a network analyzer (Agilent E8358A). All measurements were made using an output power of 0 dB, corresponding to the amplitude of the excitation current across the sample of about 1 mA. MI measurements were made after calibration and mathematical subtraction of the test fixture contributions following the well-established protocol [17]. The longitudinal MI was measured in configuration of an alternating current flowing parallel to the external magnetic field. Total impedance (Z) was measured as a function of the external magnetic field in a frequency range of the driving current frequency of 0.1 to 100 MHz.

In our previous works for MI measurements with ferrogels, we used gel pieces of particular geometry of about 0.5 g weight [5]. The measurements were strongly conditioned by the time as gel/ferrogels were rapidly changing mass, shrinking and loosing water. In the present work, we therefore used half a polymer water resistant capsule for MI measurements. We used previously synthesized stable gels/ferrogels in order to completely fill the half of the capsule with the gel/ferrogel (Figure 2).

First, the MI responses were studied by the measurements of the amorphous ribbon sensitive element itself. For the second measurements, half of the polymer capsule was situated at the center of the ribbon element (Figure 2a). The next step was to measure MI responses with the capsule filled by gel and ferrogels of the same weight but having different concentrations of the magnetic filler. As the length of the sensitive element was 18 mm the 4 mm in diameter capsule placed in a central part provided about 22% coverage of the active surface of the sensitive element. This means that the increase of the surface coverage in the future may increase the sensitivity of the detector. At the same time, there is no reason to expect a linear dependence of the sensitivity with increase of the surface coverage. The central part of the ribbon is the most appropriate position for the detection of non-uniform magnetic fields due to the absence of the contribution of the shape anisotropy near the ends of the ribbons. Measurements with the gel were used for the subtraction of the corresponding signal from the ferrogel samples in order to evaluate the average contribution of the stray fields of magnetic particles in the spatial distribution corresponding to each ferrogel case.

## 3. Results and Discussion

The idea of synthesis of ferrogels as model samples mimicking natural tissues for biosensor prototype development is a multidisciplinary approach which cannot be realized as independent steps in synthesis, material characterization and sensor prototype design. Many parameters must be optimized at a time by creating synergetic combinations of different materials working in one electronic analytical device. We therefore will comparatively analyze and discuss the most important functional properties of amorphous ribbons (MI sensitive elements), magnetic particles and, based on them, gels from the point of view of such compatibility.

### 3.1. Characterization of the Ribbons

**Figure 3:**
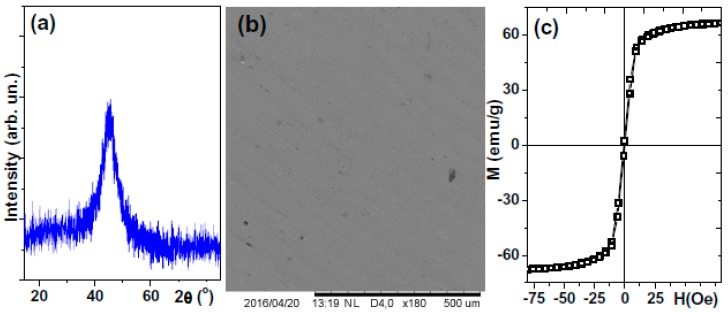
Selected properties of Co_68.6_Fe_3.9_Mo_3.0_Si_12.0_B_12.5_ amorphous ribbons: XRD patterns (**a**); surface morphology, SEM (**b**) and VSM hysteresis loop measured at room temperature (**c**).

The XRD patterns (Figure 3) of Co_68.6_Fe_3.9_Mo_3.0_Si_12.0_B_12.5_ ribbons confirmed the absence of the long-range order and the crystalline phases: a clear amorphous structure identification was possible (one very broad diffraction peak). Optical microscopy and SEM studies of the surface features show that the fabricated ribbon has a well-defined geometry (small changes of the width, Figure 1a) and a rather smooth surface with an elongated defect along the solidification direction typical for rapidly quenched ribbons (Figure 3b). The magnetostriction coefficient was very close to zero, coercivity H_c_ = 0.5 Oe and saturation magnetization M_s_ = 67 (emu/g) were defined from the shape of the hysteresis loop M(H), where M is magnetization and H is an external magnetic field applied along the ribbon axes in plane of the ribbon. H_c_ is defined as the field for where M = 0 and M_s_ is determined from the saturation of the measured magnetic moment and divided by the mass of the sample. For the sake of comparison, we include some examples of the saturation magnetization values for the amorphous ribbons of different compositions: for Co_68.5_Fe_4.0_Si_15.0_B_12.5_ M_s_ = 76 emu/g and for Co_65.9_Fe_3.5_W_3.1_Si_16.5_B_11.0_ M_s_ = 60 emu/g [44]. The observed shape of M(H) loops indicate that the effective magnetic anisotropy is a longitudinal one but a slow saturation approach can be connected with an anisotropy distribution due to frozen-in stresses and surface anisotropy contribution [44].

### 3.2. Characterization of the Magnetite and Hexaferrite Particles

The specific surface area measured by BET for both types of particles was 6.9 m^2^/g for magnetite and 3.9 m^2^/g for strontium hexaferrite samples.

**Figure 4:**
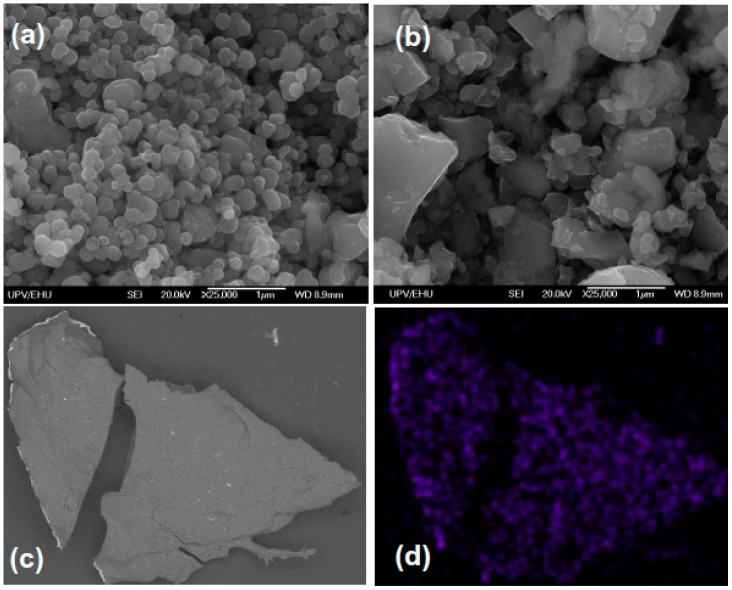
Scanning electron microscopy images of commercial “magnetite” (**a**) and “strontium hexaferrite” (**b**) magnetic particles. General view of dried piece of FG-2a ferrogel: secondary electrons SEM image (**c**) EDX analysis (Fe-Kα imaging) confirming iron presence and reasonably uniform distribution in magnetite-based dried FG-2a ferrogel sample with magnetite magnetic particles in 3.19 weight % concentration (**d**). The size of the image in (**c**,**d**) cases is 250 μm × 200 μm.

Electron microscopy images of magnetic particles used as gel fillers are shown in Figure 4. Magnetite particles are polydisperse and smoothly shaped tending to be round. Their average size is below 400 nm. Strontium hexaferrite particles are very polydisperse with particle dimensions varying from 100 to 5000 nm and irregularly shaped with sharp corners in a majority of cases. As strontium hexaferrite is produced by the sintering method with consequent milling of the product, the particles had a very mixed morphology and a pronounced degree of enhanced internal stresses.

**Figure 5:**
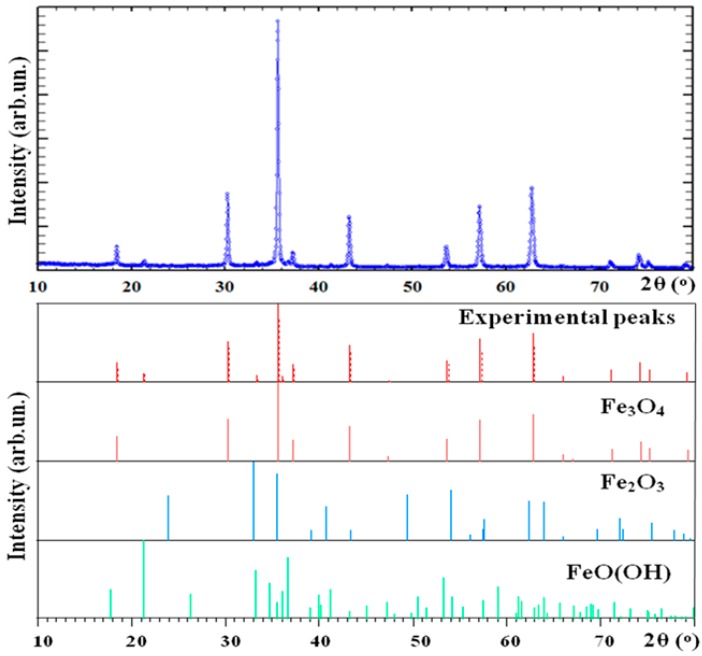
XRD patterns of commercial “magnetite” particles (top) and different phases identification as a result of peaks de-convolution using the databases identification for all observed phases (see also Table 2).

Figure 5 shows an example of XRD diffractograms of for magnetite and strontium hexaferrite SrFe_12_O_19_ powders. In all cases (see also Table 2) XRD diffractograms fitted reasonably well with the Rietveld method and crystallographic parameters were defined for all observed crystallographic phases. One can see that Fe_3_O_4_ is a main phase (94%) in the case of “magnetite” sample and SrFe_12_O_19_ is a main phase (87%) for “strontium hexaferrite” particles. Interestingly, the average sizes obtained by the XRD analysis appear to be rather smaller in comparison with the previous TEM estimation. It is understandable that due to the variety of shapes of the particles a precise size estimation is difficult in both cases. Even a simpler explanation can be given to these observations—the majority of large particles observed by SEM as individual particles are polycrystalline units having two or even more crystallographically different grains.

**Figure 6:**
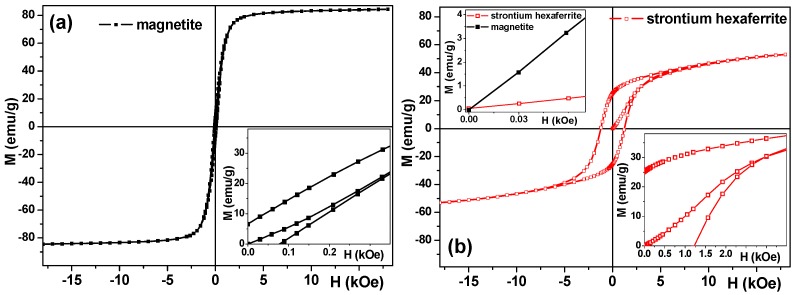
Hysteresis loops of magnetite (**a**) and strontium hexaferrite (**b**) particles. Insets in the right part of the main graphs show primary magnetization curves; inset in the left part of graph (**b**) shows primary magnetization curves of both types of particles in the small fields of interest for sensor applications.

As the next step, the magnetic properties (M(H) hysteresis loops and primary magnetization curves) of the particles measured were studied at room temperature. In the case of magnetite MPs (Figure 6a), the material almost saturates in the field of 10 kOe showing a saturation magnetization value of about 84 emu/g, i.e., the value for bulk magnetite saturation magnetization [34,45]. In the case of strontium hexaferrite (Figure 6b), the saturation was not reached even in the field of 18 kOe which is not surprising for particles with a high degree of internal stresses and irregular shapes. At the same time, the magnetization value of about 54 emu/g is quite high, also indicating that the size of the particles is large. We observed a coercive force value of about 80 Oe for magnetite and about 1200 Oe for strontium hexaferrite samples as to be expected for multidomain MPs of both types. One can also analyze the clear difference in the change of initial magnetic permeability through the primary magnetization curves–it is certainly higher for magnetite MPs, ensuring a higher magnetic moment in a low magnetic field.

### 3.3. Characterization of the Ferrogels

#### 3.3.1. Ferrogels Structure

As it is seen in Table 1, the ferrogels in the study varied in three respects: (1) the chemical nature of the magnetic particles (magnetite or strontium hexaferrite), (2) the concentration of AAm in the synthesis (0.58 M, 0.85 M and 1.1 M) and (3) the chemical origin of the thickener (guar gum or xanthan gum). The main structural feature of the polymeric matrix of a ferrogel is the mesh size of the gel network, which is the average distance between neighboring cross-links in the network. The relation between the mesh size of the network and the average size of the embedded magnetic particles determines whether the particles can freely move in the network or whether they are immobilized in it [46]. The mesh size of the ferrogel network was estimated based on the values of their swelling ratio (Table 1) using the Flory-Rehner equation for the average number of monomer units in subchains between the cross-links [47]:

(4) $$N_{C}=\frac{V_{1}(0.5\alpha^{-1}-\alpha^{-1/3})}{V_{2}(\ln(1-\alpha^{-1})+\alpha^{-1}+\chi \alpha^{-2})}$$

where *V*_1_, *V*_2_—are molar volumes of a solvent and of a polymer respectively, *χ*—is the Flory-Huggins parameter for a polymer–solvent mixture. We used *V*_1_ = 18 cm^3^/mol (water), *V*_2_ = 56.2 cm^3^/mol (PAAm) and *χ* = 0.12. The last two values were obtained by means of a quantum mechanics molecular modeling software package CAChe7.5.

As the swelling ratio diminished, if the particles were embedded in the gel matrix we took blank gels for the estimation of the upper limit for the mesh size of the polymeric network. Equation (6) gave the average number of monomer units in linear sub-chains N_C_ = 240, 1470 and 11,360 in respect to the polymeric network based on 1.1 M, 0.85 M and 0.58 M AAm concentration. The average distance between the cross-links, i.e., the mesh size, was then estimated using the well-known equation for the mean square end-to-end distance of the random Gaussian coil with hindered rotation [48]. It gave the upper limit for the mesh size: 5 nm, 12 nm and 33 nm for the mentioned set of AAm concentration. If we compare the mesh size to the average size of magnetite or strontium hexaferrite magnetic particles (see Figure 4), we will see that the particles are immobilized in the ferrogel network, which in this case might be considered as an elastic continuous medium for the magnetic filler. Polysaccharides, like any biopolymers, are complex multilevel systems with complex conformational and aggregative behavior, which is not fully understood even in individual solutions. For example, in our recent work one particular aspect of this behavior was discussed [49]. In addition, we should point out that, despite the chemical similarity of polysaccharides to each other, they demonstrate significant and poorly understood differences in behavior. The systems analyzed in the present work are even more complex including polyacrylamide mesh and filler. It is impossible to provide sufficient understanding of all these details at this time. We did focus on the polymer peculiarities of polysaccharides but simply used them as thickeners.

The weight fraction of magnetic particles in ferrogels was a dependent variable as it was related to the swelling ratio of the ferrogel according to Equation (2). The value of the MPs’ fraction also determines the magnetic moment of the ferrogel. For magnetic biosensing, low concentrations (below 5 wt %) are usually employed in order to avoid an interaction process and interferences which would complicate the magnetization of the stray fields of the ensemble. Selected concentrations are therefore useful for comparative studies of both mechanical and magnetic properties.

#### 3.3.2. Young Modulus of Ferrogels

**Figure 7:**
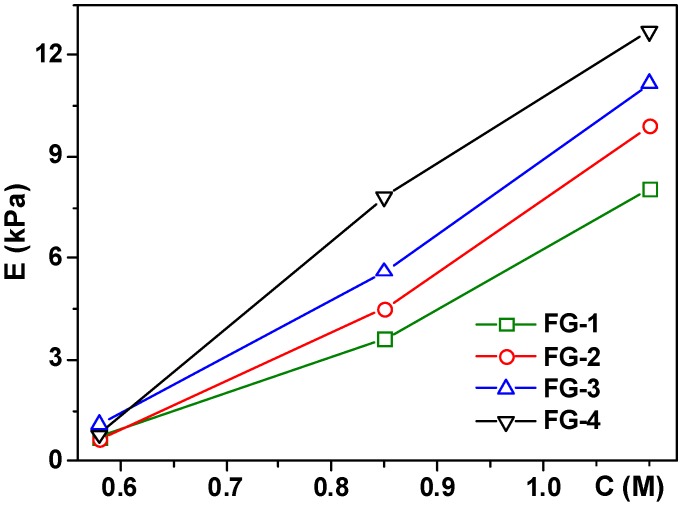
Young modulus of ferrogels with different concentration of acrylamide (C) taken in the synthesis of gel matrix for different FG series: FG-1 filled with magnetite if the thickener was guar gum and FG-2 if the thickener was xanthan gum, filled with strontium hexaferrite FG-3 (thickener–guar gum) and FG-4 (thickener–xanthan gum). Weight fraction ranges of magnetic particles were 2.59–5.20 wt % for FG-1, 0.77–3.19 wt % for FG-2, 1.31–3.70 wt % for FG-3 and 0.77–3.33 wt % for FG-4. Lines are for eye-guide only. C-concentrations were as high as 1.1 M, 0.85 M and 0.58 M aiming to provide gels with varying elasticity.

As the polymeric matrix constitutes the continuous phase of a ferrogel, its mechanical strength is mostly influenced by the composition of a polymeric network. In this study, the density of the polymeric network was changed by varying the AAm concentration in the synthesis. At a low AAm monomer concentration in the synthesis the polymeric matrix contained less polymeric chains than at a high AAm concentration. Figure 7 presents the dependence of the Young modulus of the ferrogels with different thickeners on the concentration of AAm monomer taken in the synthesis. The Young modulus of ferrogels strongly depends on the AAm concentration. With a two-fold increase in concentration (from 0.58 to 1.1 M) the modulus increased ca ten-fold—from 1 kPa up to 8–12 kPa depending on the ferrogel series. In the studied concentration range of AAm, the dependence of the modulus is almost linear. It means that the influence of the density of polymeric network on the mechanical strength dominates over the influence of other composition variables (Table 1). Meanwhile, the influence of the origin of a thickener and the chemical nature of magnetic material is still noticeable.

Concerning the origin of the thickener, xanthan gum provides higher values of the Young modulus than guar gum (compare FG-1 with FG-2 and FG-3 with FG-4 in Figure 7). It means that the addition of polysaccharide to the system is not exclusively restricted to the increase of elasticity. As mentioned above, polysaccharides form weak physical networks in solution [50]. Thus, introducing of a polysaccharide into the synthesis of a ferrogel results in the mixing of two types of polymeric networks–the chemical one provided by the cross-linked polyacrylamide and the physical one comprised of the aggregated polysaccharide macromolecules. Such systems are known as the semi-interpenetrating networks (semi-IPN) [49,50] and their properties depend on the nature of both networks.

As for the nature of the magnetic material, ferrogels FG-3 and FG-4 filled with strontium hexaferrite particles have a systematically larger value of the Young modulus than ferrogels filled with magnetite particles (compare FG-1 with FG-3 and FG-2 with FG-4 in Figure 7). Most likely, the dependence of the modulus on the magnetic material stems from the specific features of the adhesion of network polymeric subchains on the surface of the particles. Apparently, the adhesion of the gel network to the surface of strontium hexaferrite is stronger than the adhesion to the surface of magnetite. It makes the modulus of strontium hexaferrite based ferrogels (FG-3 and FG-4) higher than the modulus of magnetite based ferrogels (FG-1 and FG-2).

#### 3.3.3. Deformation of Ferrogels in a Uniform Magnetic Field

**Figure 8:**
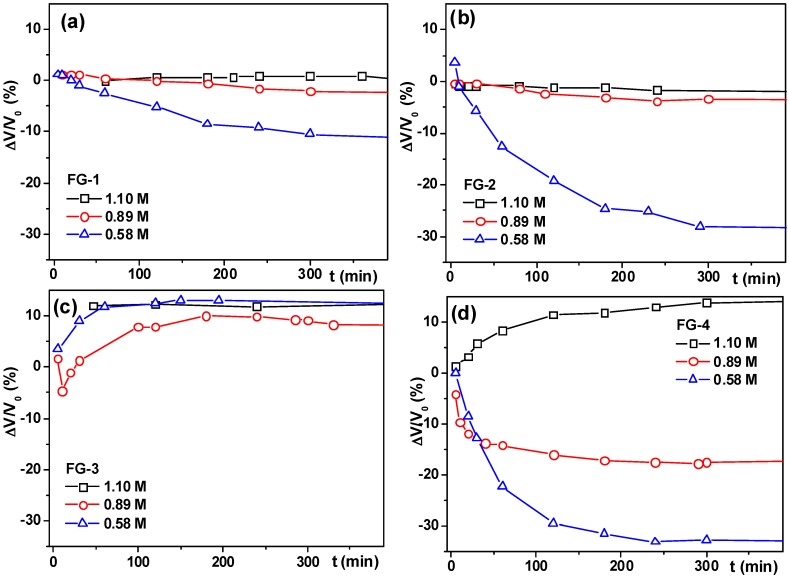
Time dependence of the volume deformation of ferrogels in the uniform magnetic field 0.420 T. (**a**) FG-1 series, (**b**) FG-2 series, (**c**) FG-3 series and (**d**) FG-4 series. Lines are for eye-guide only. FG-1 series filled with magnetite if the thickener was guar gum and FG-2 if the thickener was xanthan gum, filled with strontium hexaferrite FG-3 (thickener–guar gum) and FG-4 (thickener–xanthan gum). Weight fraction ranges of magnetic particles were 2.59–5.20 wt % for FG-1, 0.77–3.19 wt % for FG-2, 1.31–3.70 wt % for FG-3 and 0.77–3.33 wt % for FG-4. The legend of the plot gives the concentration of AAm in the synthesis of ferrogels, which determines the Young modulus.

Figure 8 shows how the volume of a ferrogel sample changes if it is placed in a uniform magnetic field of 4.2 kOe. As mentioned in the Methods section, the measured values were the diameter and the height of the cylindrical sample in the field. It was found that in all cases both the height and the diameter changed in the same way. If the gel dimension along the field (the diameter of a gel) increased so did the dimension across the field (the height of a gel). The same can be observed concerning the decrease in diameter and height. We have never observed opposite changes like the decrease in diameter but an increase in the height. Due to such similarity of the deformation along the field and across the field these data were combined to give the total volume change of a gel in field. The relative volume change was calculated by the following equation:

(5) $$\frac{V}{V_{0}}=\frac{hD^{2}}{h_{0}D_{0}^{2}}$$

where *h*_0_ and *D*_0_ are the height and the diameter of a gel without the applied field, *h* and *D* are the values if the field was applied for a certain period of time. The curves in Figure 8 give the relative increase or the decrease (in %) of the gel volume in the field. If the gel contracts in the magnetic field, the relative volume change is negative, if the gel swells–the volume change is positive.

There are two basic trends in the curves shown in Figure 8. One is the decrease of gel volume in the field (contraction), another is the opposite–the increase of the volume i.e., swelling. Both trends were reported in an earlier work for ferrogels based on the polyelectrolyte matrix of the copolymer of acrylamide and potassium acrylate [43]. The prevalence of a trend depends on the composition variables of the ferrogel. In the case of the FG-1 and FG-2 series based on magnetite, the contraction trend prevails in all compositions (Figure 8a,b). There is the obvious influence of the density of the polymeric matrix of the ferrogel. Ferrogels with a dense matrix provided by a high concentration of AAm in the synthesis, show no or a very small contraction. Weak ferrogels with the lowest concentration of AAm show a much stronger response to the field. Such a dependence is reasonable as the concentration of AAm governs the Young modulus of the ferrogels (Figure 7). At the same time, the influence of the polysaccharide does not follow the trend in the modulus. As shown in Figure 7, gels with a xanthan physical network are stronger and have higher modulus than ferrogels with a guar physical network. Despite this, the response of the FG-2 (“xanthan”) series to the field is stronger than the response of the FG-1 (“guar”) series. The maximal contraction obtained for magnetite based ferrogels was 30% of contraction for the gel synthesized in 0.58 M AAm with xanthan physical network.

The positive feedback of the xanthan physical network on the deformation of ferrogels in the field is also present in strontium hexaferrite based gels FG-3 and FG-4. However, the trends are more complex. Contraction, swelling and their combination are observed for these gels. Gels of FG-3 series in general swell in the magnetic field. However, in the case of FG-3-0.85 gel, small initial contractions were observed at the early stages of field application. Gels of FG-4 series do not show a uniform pattern. Gel FG-4-1.10 with a high compression modulus swells up to 10% in the field like gels of the FG-3 series. At the same time, gels FG-4-0.85 and FG-4-0.58 with a lower Young modulus contract in the field like gels of FG-1 and FG-2 series. The maximal contraction is around 30% like in the FG-2 series.

We are not ready at this point to give an explanation as to why the applied external field causes changes in the ferrogel volume and the volume changes with respect to time. For now, we consider them experimental facts. In different systems both contraction and expansion were observed. In principle, the explanation of this behavior is related to the minimization of the free energy of particle interaction. It does this by changing the degree of swelling of the matrix. The degree of swelling according to modern concepts depends on at least four different thermodynamic forces, plus the forces of magnetic interaction of particles, the equations for which are also not completely defined, since they strongly depend on their mutual arrangement. All this makes the description extremely complex and uncertain at present. What is clear is that the modulus of the gel does not exceptionally govern the magnetically induced deformation. It is also clear that several structural parameters are missing from the analysis. Among them certainly are the aggregation degree of magnetic particles and the structure of aggregates. Unfortunately, these features are very difficult to characterize in such systems as a cross-linked gel. There was an experimental observation [51,52,53] that the linear dimension of ferrogels based on siloxane resins along the field lines increased if the magnetic particles were uniformly distributed in the polymeric matrix and it decreased if the magnetic particles in the synthesis were pre-aligned in the direction of the field. It means that the formation of aggregates of magnetic particles in the gel is crucially important for its deformation in the uniform magnetic field.

### 3.4. Magnetic Properties of Ferrogels

**Figure 9:**
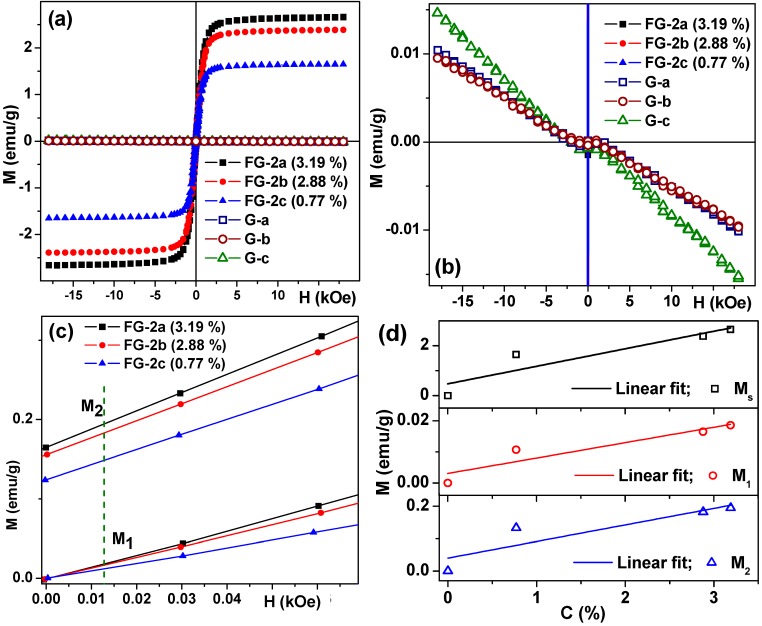
Magnetic properties of gels and ferrogels with magnetite particles. M(H) loops of gels/ferrogels in the field range in which the saturation tendency for ferrogels is observed (**a**). For FG-2 series the thickener was xanthan gum. M(H) loops of gels/ferrogels in low magnetic moment values scale for which the lack of the saturation tendency for gels is appreciated (**b**). Primary magnetization curves and close to remanence behavior for ferrogels: M_1_ (for primary magnetization curve) and M_2_ (for approaching remanence) values of the magnetic moments of ferrogels, green dashed line indicates magnetic field close to 12 Oe (**c**). Concentration dependence of M_s_ and M_1_, M_2_ parameters for FG-2 ferrogels (**d**).

The magnetic properties of xanthan containing gels were studied in detail. Figure 9 shows hysteresis loops of magnetite-based gels and ferrogels with different concentrations of MPs. The gel matrix contributes very little to the total magnetic signal but this diamagnetic contribution can be very precisely measured by VSM and extracted from the ferrogel signal if necessary. Unfortunately, the M(H) loops for gels had a very small ferromagnetic contribution, corresponding to impurities but this contribution was very similar for gels with different amounts of thickener and it was at least 4 orders of magnitude smaller in comparison with ferromagnetic contribution of magnetic particles (Figure 9b). The values of saturation magnetization of ferrogels show a linear dependence on the magnetic filler concentration, as expected for materials with a low concentration of MPs (Figure 9d). Although the saturation magnetization M_s_ is a very important parameter for the characterization of magnetic materials and the understanding of their basic properties, in many technological applications and biomedicine, magnetic materials are not used in the saturated state. It is simply because the application of a high magnetic field is costly, difficult to apply (a high field) in a compact device and it is subject to special biomedical regulations [54].

Let us analyze the magnetization behavior in the positive fields starting from the demagnetized state (primary magnetization curve) and approaching zero field after saturation, i.e., approaching the state of remanence (Figure 9c). In order to create an efficient detector of the stray fields of the magnetic labels, the parameters of the two magnetic materials (amorphous ribbon and magnetic particles in the present case) must be adjusted to each other: the work interval of high sensitivity of the MI sensitive element should overlap with the field interval where magnetic particles have sufficiently high magnetic moments in order to create a sizeable magnetic field. Coming back to the M(H) hysteresis loop of the amorphous ribbon, one can see that 12 Oe value is a critical field related to an effective magnetic anisotropy in which high magnetoimpedance sensitivity can occur. Selecting two characteristic values M_1_ (for primary magnetization curve) and M_2_ (for approaching remanence) for a 12 Oe external magnetic field, we can plot their concentration dependence for FG-2 ferrogels, which also appears to be linear. Interestingly, the M_1_ values corresponding to primary magnetization curves are of about two orders of magnitude smaller in comparison with M_s_ and one order of magnitude smaller in comparison with M_2_ corresponding values.

Figure 9d shows the concentration dependence of M_s_, M_1_ and M_2_ parameters for FG-2 ferrogels. The first experimental point corresponds to the case of gel with no magnetic particles included. As one can estimate from the hysteresis loop of corresponding gels, the magnetic contribution of the gel without NPs is at least two orders of magnitude smaller in comparison with contribution of ferrogels.

**Figure 10:**
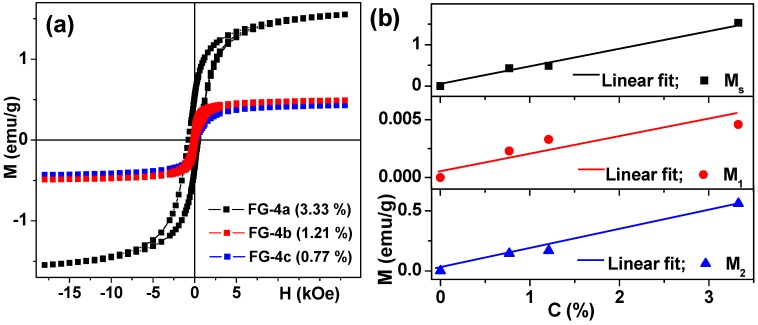
Magnetic properties of ferrogels with strontium hexaferrite particles (see also Table 1). M(H) loops of ferrogels in wide field range in which the saturation tendency for ferrogels is observed (**a**). For FG-4 series the thickener was xanthan gum. Concentration dependence of the saturation magnetization and M_1_, M_2_ parameters for FG-4 ferrogels: M_1_ (for primary magnetization curve) and M_2_ (for approaching remanence) values of the magnetic moments of ferrogels (**b**).

Figure 10 shows hysteresis loops of strontium hexaferrite-based ferrogels with different concentrations of MPs. Again, the gel matrixes contribute very little to the total magnetic signals but they can be carefully extracted from the ferrogel signals. The same as in the case of ferrogels with a magnetite filler, the values of saturation magnetization of ferrogels (Figure 10b) and two characteristic values M_1_ (for primary magnetization curve) and M_2_ (for approaching remanence) for a 12 Oe external magnetic field show linear dependence on the magnetic filler concentration.

An interesting observation can be made at this point: M_2_ values for strontium ferrite ferrogels of the same concentration are characterized by about twice higher magnetic moments (low field case of remanence approach) in comparison with magnetite based gels, indicating similar or better opportunities for the detection by a magnetic field sensor of strontium hexaferrite MPs based ferrogels. Coming back to the magnetic properties of the particles of magnetic fillers (Figure 6), one can notice that the magnetization value in the field of 18 kOe is much higher in the case of magnetite particles. But the value of magnetization in the field of 12 Oe is larger for strontium hexaferrite MPs ferrogels due to magnetic hysteresis. For ferrogels with nanosized particles of a magnetic filler, due to the superparamagnetic state of the nanoparticles the simple following relation is expected: the higher the value of saturation magnetization, the higher the magnetic moment value in a small field [34]. For large scale MPs above the superparamagnetic-ferrimagnetic transition, this relation is not necessarily valid—M_s_ is higher for magnetite particles (and gels on their basis) but M_2_ values are higher for strontium hexaferrite MPs based gels.

We would like to emphasize that the idea of using large scale MPs above the superparamagnetic-ferrimagnetic transition was not only stimulated by the fact that the magnetization signals at low field (M_1_ and M_2_) in the case of nanoparticles are too low for the detection of very small concentrations of them in ferrogels. In our previous work, we have demonstrated that the detection of 20 nm iron oxide nanoparticles in ferrogels in the same concentration interval was possible [5]. The main idea was to improve the detection limit following the well-known law at nanoscale–the larger the size, the higher the saturation magnetization [34]. At the same time MPs above the superparamagnetic-ferrimagnetic transition tend to aggregate and show a much more complex magnetic behavior, as we have demonstrated above. Synthesis of ferrogels with large particles can be a good solution to some extent, preventing coarse particle aggregation especially under the application of an external magnetic field. Encapsulated particles can be delivered to the point of therapy using a magnetic field for different purposes.

For example, the possibility of biomedical applications of larger nanoparticles up to 300 nm size is being discussed [27]. In solid tumors due to angiogenesis, i.e., the growth of new blood vessels, irregular gaps much bigger than the ones in healthy blood vessels are present. Nanoparticles of less than 300 nm in diameter can pass through such big gaps and accumulate in the tumors. Apart from field assisted delivery it is necessary to control the number of magnetic particles accumulated at a certain point. We propose to use a magnetic field sensor based on the magnetoimpedance effect for this purpose. In order to demonstrate the validity of the concept we have prepared ferrogel samples with large magnetic particles, systems mimicking tumor tissue with embedded particles.

### 3.5. Magnetoimpedance Measurements in Configuration of Biosensor Prototype

**Figure 11:**
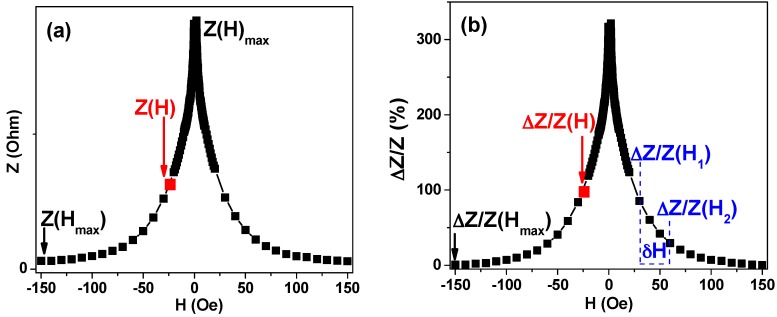
Magnetoimpedance related definitions for magnetoimpedance ratio ∆(*Z/Z*)(H) (**a**) and MI ratio sensitivity S(∆(*Z/Z*)(H) calculations (**b**).

The MI ratio was calculated as follows:

(6) $$\frac{\Delta Z}{Z}=100\times \frac{Z(H)-Z(H_{\max})}{Z(H_{\max})}$$

where *H*_max_ = 100 Oe. For MI frequency dependence analysis, the changes of the maximum value of the total impedance Δ(*Ζ/Ζ*)_max_ was appropriate. MI sensitivity for total impedance and its real part were denominated as S(Δ*Z*/*Z*) and *S*(Δ*R*/*R*) accordingly:

(7) $$S\left(\frac{\Delta Z}{Z}\right)=\frac{\delta (\Delta Z/Z)}{\delta H}$$

where *δ*(Δ*Z*/*Z*) is the change in the total impedance or real part GMI ratio for the magnetic field increment *δ*(*H*) = 0.1 Oe. Figure 11 helps to understand the MI related definitions. Primarily the impedance modulus is calculated in Ohms from the experimentally measured voltage drop. *Z*(*H*_max_) is the impedance in the maximum field (Figure 11a). For magnetic material showing strong MI effect generally speaking *Z*(*H*_max_) ≠ *Z*(*H*). This dependence is easier to understand analyzing exact numbers for MI ratio: *Z*(*H*_max_) = 0 for the field *H* = 150 Oe but *Z*(*H*) = 95 Oe for the field *H* = 25 Oe (compare points for black and red arrows). The blue font part helps to represent the concept of sensitivity: let us select two values of the magnetic field (*H*_1_ and *H*_2_). $S\left(\frac{\Delta Z}{Z}\right)=$ (∆(*Z/Z*)(*H*_1_)−∆(*Z/Z*)(*H*_2_))(*H*_2_−*H*_1_) corresponds to the MI sensitivity in the field interval of H_1_ to H_2._ Here we used a higher field increment (not *δ*(*H*) = 0.1 Oe) just for simplicity.

The experimental error in determining the impedance was within 1%. Direct current resistivity (R_DC_) was also measured in all cases. MI measurements were made at room temperature.

**Figure 12:**
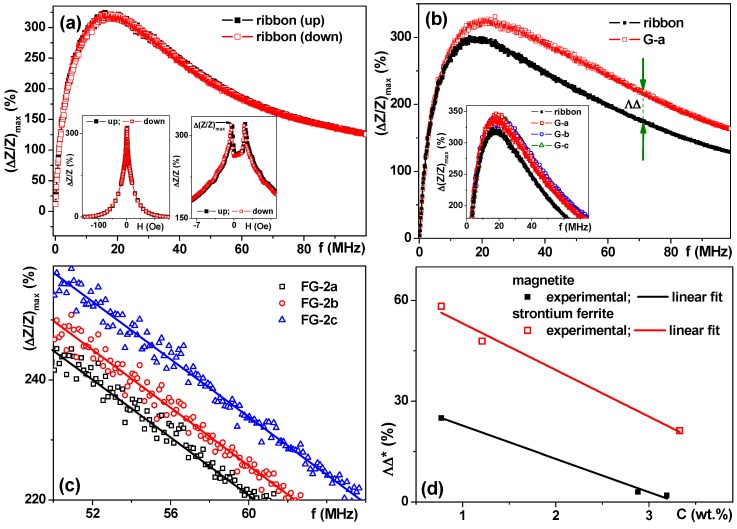
Frequency dependence of (Δ*Z/Z*)_max_ ratio for Co_68.6_Fe_3.9_Mo_3.0_Si_12.0_B_12.5_ rapidly quenched amorphous ribbon. Insets show field dependence of Δ(*Z/Z*) ratio for 15 MHz frequency: left inset–is given for large field interval and right inset is given for low magnetic fields (**a**). Frequency dependence of (Δ*Z/Z*)_max_ ratio for amorphous ribbon without and with G-a gel. Green arrows explain definition of ΛΔ parameter. Inset shows better resolution image for (Δ*Z/Z*)_max_ ratio for amorphous ribbon without and with G-a, G-b and G-c gels (**b**). Selected region of frequency dependence of (Δ*Z/Z*)_max_ ratio for amorphous ribbon with FG-2 ferrogels, the thickener was xanthan gum (**c**). Concentration dependence of the ΛΔ* = ΛΔ(gel)–ΛΔ(ferrogel) difference for FG-2 and FG-4 ferrogels for frequency f = 60 MHz (**d**).

Next, we comparatively studied MI responses of the amorphous ribbon sensitive elements in the presence of gels and FG-2 and FG-4 ferrogels (Figure 12). First of all, the MI of the sensitive element was measured in a frequency range of 1 to 100 MHz without any gel/ferrogel. The shape of (Δ*Z/Z*)_max_ curve is typical for Co-based amorphous ribbons [55,56] which can be described in the frame of classic electrodynamics and field dependence of transverse dynamic magnetic permeability in a condition of strong skin effect [56,57]. Figure 10a shows two MI (Δ*Z/Z*)_max_ curves: the “up”–(Δ*Z/Z*)_max_ curve was measured in the field starting from saturation in the maximum negative external magnetic field; the “down”–(Δ*Z/Z*)_max_ curve was measured in the field starting from saturation in the maximum positive external magnetic field. One can clearly see that both curves overlap providing high quality measurements.

Insets in Figure 12a help to understand better the definition of the (Δ*Z/Z*)_max_ value. The field dependence of the Δ*Z/Z* ratio is again typical for the MI response of amorphous ribbons with longitudinal magnetic anisotropy—one-peak shape MI response [37], as we could expect from the shape of the hysteresis loop of the ribbon (Figure 2c). However, a higher resolution representation shows a fine structure of the Δ*Z/Z*(H) curve showing two peaks. The existence of two peaks can be explained by the contribution of the surface anisotropy by adding a small transverse magnetic anisotropy component [37].

Here we can briefly discuss the concept of sensitivity of the magnetic field sensor with respect to the applied magnetic field. There are different field regions with close to linear Δ*Z/Z*(H) dependence: in a small magnetic field for 1.4 Oe <|H|< 1.9 Oe, $S\left(\frac{\Delta Z}{Z}\right)=100\%/\mathrm{Oe}$; and in the high magnetic field for 6 Oe <|H|< 15 Oe, $S\left(\frac{\Delta Z}{Z}\right)=8\%/\mathrm{Oe}$. Despite the fact of extremely high low field MI sensitivity, the linearity interval is very narrow (0.5 Oe) making it not useful for practical applications. The work interval in the high magnetic field is wide (9 Oe). We will show below that even such a modest MI sensitivity as 8%/Oe is sufficient for the measurement of ferrogel concentration dependence using this magnetic field sensor.

Figure 12b shows a frequency dependence of the MI ratio of Co_68.6_Fe_3.9_Mo_3.0_Si_12.0_B_12.5_ amorphous ribbon without and with G-a gel piece (see also Figure 2b). The effect of the gel presence is clearly manifested by the increase of (Δ*Z/Z*)_max_ ratio. In order to quantify the observed difference, let us use the ΛΔ parameter defined as ΛΔ = (Δ*Z/Z*)_max_(for gel) − (Δ*Z/Z*)_max_(for ribbon) at f = const which was useful for a rather wide frequency range of 20 to 100 MHz.

Inset Figure 12b shows the frequency dependences of the MI ratio of amorphous ribbon without and with different gels. Although gel responses are close to each other, they are not exactly identical, indicating the differences in dielectric properties of the gels. On the one hand, this fact makes the extraction of the stray field responses in the case of ferrogels more complicated. On the other hand, it opens the possibility to use magnetoimpedance spectroscopy as a new technique useful for the evaluation of the properties of even non-filled gels in the future. The stray field created by the single uniformly magnetized particle can be calculated using a pure dipole model [14] for two possible geometries [58]: with magnetization of the bead either perpendicular, or parallel to the plane of the sensitive element of the sensor. In literature, there are at least four reported bead detection configurations [58,59,60], in which bead magnetizing field is perpendicular, parallel or under a certain angle to the sensor plane and with or without modulation across the sensitive element. In the case of ferrogels with rather large particles, simple modeling is not applicable and comparative analysis of the magnetic pre-history and MI responses become crucially important.

Figure 12c shows the frequency dependence of the MI ratio of the amorphous ribbon without and with FG-2 ferrogels (see also Figure 2b). The effect of ferrogel presence is more complex compared to the gel response. Although the corresponding gel presence leads to an increase of (Δ*Z/Z*)_max_ ratio, the FG response becomes close to the (Δ*Z/Z*)_max_ ratio of the ribbon indicating the existence of at least two contributions with different tendencies. Even so, the FG-2 responses measured directly for FG-2 with different filler concentrations (C, in weight %) show the following tendency: the higher the C value the lower the (Δ*Z/Z*)_max_ ratio.

Next, we recalculated the MI values measured for the samples of gels and ferrogels of two types-with magnetite (FG-2) and strontium hexaferrite (FG-4) fillers. It is worth mentioning, that all of the samples for these tests had the same mass (0.070 mg). The DΔ* = DΔ(gel) − DΔ(ferrogel) parameter reflected the contribution of the stray fields of magnetic particles. Two observations are important. The first one is that in both cases DΔ* parameter shows linear decay with the increase of the concentration of the magnetic filler. The second one is that in the case of strontium ferrite a slightly higher sensitivity for the concentration dependence was measured.

At first glance, a higher sensitivity in the case of strontium ferrite MPs seems to be contradicting our previous experience and the existing understanding of the origin of MI sensitivity, namely the disturbance of the external magnetic field by the stray field of magnetic particles. In fact, the detection of the non-single domain (not superparamagnetic particles) requires a different approach. The absolute value of the magnetic moment of superparamagnetic nanoparticles in a certain field does not depend on the magnetic history of superparamagnetic MNPs due to the absence of magnetic hysteresis. For big particles, it is not correct and the value of the magnetic moment in certain magnetic fields depends on the magnetic history and can be very different (Figure 9d and Figure 10b). Magnetite MPs have higher M_s_ value than strontium hexaferrite MPs but the M_2_ parameter defined above is the magnetic moment governing the value of the stray fields of large MPs with non-zero coercivity and it is higher for strontium hexaferrite MPs resulting in a slightly higher MI sensitivity to the concentration dependence.

Of course, any dependence on magnetic history can be seen as a disadvantage for the detection process but the concept is clear and the technological solution can be simple: short period application of a reasonably high external magnetic field prior to the detection process, which can be simply a field created by the permanent magnet.

## 4. Conclusions and Outlook

Gels and ferrogels with a polymeric network of polyacrylamide were synthesized by radical polymerization of monomeric acrylamide (AAm) in a water solution at three levels of concentration: 1.1 M, 0.85 M and 0.58 M providing gels with varying elasticity. Their mechanical and magnetic properties were comparatively analyzed. Ferrogels filled with strontium hexaferrite particles have a systematically larger value of the Young modulus than ferrogels filled with magnetite, most likely due to the specific features of the adhesion of polymeric subchains of the network on the surface of the particles. Apparently, the adhesion of the gel network to the surface of strontium hexaferrite is stronger than the adhesion to the surface of magnetite.

There are two possible trends in the deformation process of ferrogels: the decrease of gel volume in the field or the increase of the volume, i.e., swelling. The prevalence of a trend depends on the composition variables of the ferrogel. In the case of the series based on magnetite, the contraction trend prevails in all compositions: ferrogels with a dense matrix provided no or very small contraction. Weak ferrogels with the lowest concentration of AAm show a much stronger response to the field as the concentration of AAm governs the modulus of the gel. At the same time the influence of the polysaccharide does not follow the trend in modulus. Physical networks with xanthan are stronger and have higher modulus than ferrogels with guar physical networks. Despite this, the response of the “xanthan” series to the field is stronger than the response of the “guar” series. The maximum contraction obtained for magnetite based ferrogels was 30% of contraction for the gel synthesized in 0.58 M AAm with xanthan physical network. The positive feedback of the xanthan physical network on the deformation of ferrogels in the field is also present in strontium hexaferrite based gels. However, the trends are more complex. Contraction, swelling and their combination are observed in these gels. The maximum contraction is around 30% like in magnetite-based series.

Magnetic properties of magnetite and strontium hexaferrite particles and gels and “xanthan” series of ferrogels were systematically studied and comparatively analyzed. The saturation magnetizations of the particles were in both cases quite close to the bulk values, quite in accordance with structural peculiarities and the sizes of the particles.

We have designed a small magnetoimpedance biosensor prototype with Co_68.6_Fe_3.9_Mo_3.0_Si_12.0_B_12.5_ rapidly quenched amorphous ribbon based element aiming in the future to develop a sensor working with a disposable stripe sensitive element. The developed protocol allowed measurements of the concentration dependence of magnetic particles in magnetite and strontium hexaferrite ferrogels with xanthan using the MI effect. We have discussed the importance of magnetic pre-history and demonstrated importance of remnant magnetization in the detection process.

We have shown the possibility of detecting large magnetic particles using an amorphous ribbon MI sensitive element. Our main result is that we were able to create such model samples mimicking natural tissue. They can be viewed as reliable substitutes for living tissues. This study opens new possibilities for the development of biosensors for the detection of the particles in large volumes. More systematic studies are necessary in order to improve this simple prototype up to the level requested by biomedical applications and research other types of ferrogels and biocomposites but the direction seems to be promising.

## Figures and Tables

**Table 1:** Description of the synthesized gel and ferrogel samples: composition and swelling ratio.

G/FG Series	Magnetic Particles	Concentration of AAm (M)	Thickener	Swelling Ratio, α	Weight fraction of Magnetic Particles, ϑ (%)
FG-1a	Fe_3_O_4_	1.10	Guar	10	5.20
FG-1b	Fe_3_O_4_	0.85	Guar	15	4.12
FG-1c	Fe_3_O_4_	0. 58	Guar	27	2.59
FG-2a	Fe_3_O_4_	1.10	Xanthan	17	3.19
FG-2b	Fe_3_O_4_	0.85	Xanthan	21	2.88
FG-2c	Fe_3_O_4_	0. 58	Xanthan	93	0.77
FG-3a	SrFe_12_O_19_	1.10	Guar	15	3.70
FG-3b	SrFe_12_O_19_	0.85	Guar	51	1.24
FG-3c	SrFe_12_O_19_	0. 58	Guar	54	1.31
FG-4a	SrFe_12_O_19_	1.10	Xanthan	16	3.33
FG-4b	SrFe_12_O_19_	0.85	Xanthan	52	1.21
FG-4c	SrFe_12_O_19_	0. 58	Xanthan	92	0.77
G-a	-	1.10	Xanthan	31	0
G-b	-	0.85	Xanthan	90	0
G-c	-	0. 58	Xanthan	303	0

**Table 2:** Results of XRD analysis of the most intensive peaks for commercially available “magnetite” and “strontium hexaferrite” particles.

Particles Type	Phases	2θ (˚)	FWHM_av_ (˚)	Intensity	Phases Content (%)	D_XRD_ (nm)
“Magnetite”	Fe_3_O_4_	35.631	0.164	2799.33	94	130
	Fe_2_O_3_	33.354	0.209	27.61	1	75
	FeO(OH)	21.369	0.216	75.64	5	70
“Strontium hexaferrite”	SrFe_12_O_19_	34.349	0.136	712.19	87	230
	Fe_2_O_3_	33.319	0.262	166.16	13	50
